# Effect of Drought on the Future Sugar Content of Wine Grape Varieties till 2100: Possible Adaptation in the Hungarian Eger Wine Region

**DOI:** 10.3390/biom13071143

**Published:** 2023-07-18

**Authors:** László Lakatos, Zoltán Mitre

**Affiliations:** 1 Department of Environmental Science and Landscape Ecology, Eszterházy Károly Catholic University, 1. Eszterházy tér, H-3300 Eger, Hungary; 2 Institute of Geography and Earth Sciences, Faculty of Sciences, University of Pécs, 6 Ifjúság útja, H-7624 Pécs, Hungary

**Keywords:** climate change, dryness index, the sugar content of wine grapes, IPCC periods, simulated deficit irrigation, optimal dryness demand

## Abstract

The most significant risk for viticulture is that the sugar content of the grapes will increase in the future due to rising temperatures. As a result, it will be possible to produce wines with increasing alcohol content in the future. Excessively high alcohol content can significantly reduce the wines’ marketability and viticulture’s profitability. Our study seeks to answer how the expected drought in the Southern and Central regions of Europe will affect the future change in the sugar content of grapes. The degree of dryness was examined using the dryness index in the study. Finally, it was analyzed how the dryness index affects the past and future occurrence of maximum sugar content for six grape varieties. The probability of the occurrence of maximum sugar content for most vine grape varieties will decrease in the near future. However, in the distant future, there is a likelihood that the occurrence of maximum sugar content will increase again. If we can maintain the DI ≥ −10 condition with regulated deficit irrigation, the probability of the occurrence of maximum sugar content may decrease significantly in the near future. Ensuring moderate dryness is the only way to achieve the proper sugar content.

## 1. Introduction

The grape (*Vitis vinifera* L.) grows best in areas with a Mediterranean climate. It particularly values abundant rainfall during the dormant period and tolerates drought during the growing season. Grapes are grown in the Mediterranean and areas with a continental climate up to 50 degrees latitude [1]. In these areas, which are currently characterized by “cool” climatic conditions, the significant diurnal temperature range at ripening is particularly favorable for cultivating quality wine grapes. Because of climate change effects, the dryness will increase not just in the Mediterranean region of Europe but also in the Carpathian basin [2]. These changes will provide even more favorable climatic conditions for quality wine production in the future than the current traditional wine-growing areas at lower latitudes. Based on climate models, the severity and frequency of drought in most famous vineyards will increase in the future, meaning heat and drought stress will affect these areas more and more in the future, thus jeopardizing the quality of wine grape production. The biggest fear of growers is that due to rising temperatures and more frequent droughts, the sugar content of grapes will increase, which will increase the alcohol content of wines. Due to the higher alcohol content, the fermentation process changes, resulting in a wine with a different taste and character. In the case of sensory evaluations, these wines with an alcohol content higher than necessary usually seem unbalanced, with an upset to the harmonic sugar/acid ratio. The onset of drought associated with warming will accelerate the increase in the sugar content of berries in the future. Drought is a complex concept that occurs when several factors go hand in hand. It can be stated that the sugar content of berries at the time of ripening is influenced not only by drought or water stress but also by the grape genotype, vineyard temperature, and canopy management, all of which affect the magnitude of sugar content [3].

Unfavorable environmental conditions can cause separate heat and water stress in grapevines, which affect the grape’s internal parameters such as sugar and acid content, the development of phenolic compounds, and yield quantity. Naturally, these effects often occur in combination. However, according to [4], heat stress had a more significant impact on gene expression in grape berries than water stress.

In this study, we aimed to examine the effects of drought and the dryness index (DI), which quantifies drought, on the development of sugar content in ripening grape berries.

Drought stress causes a morphological, physiological and biochemical response in grape wine plants [5]. Heat stress and severe drought together may compromise photosynthesis, causing a source–sink imbalance in the grapes, obtaining low-quality musts for vinification [6].

Severe drought can cause a loss of turgor and cause xylem cavitation formation, which can lead to leaf fall and even grape death [7]. The water deficit reduces berry size and increases the levels of soluble sugars, total phenols and anthocyanins [8]. The glycosylated volatile organic compounds of the berries show a positive correlation with the severity of water stress prior to veraison [9].

Due to the likely future occurrence of increasingly frequent and severe droughts, a proper irrigation strategy is essential for obtaining high-quality berries and wines [9,10,11].

**Figure 1:**
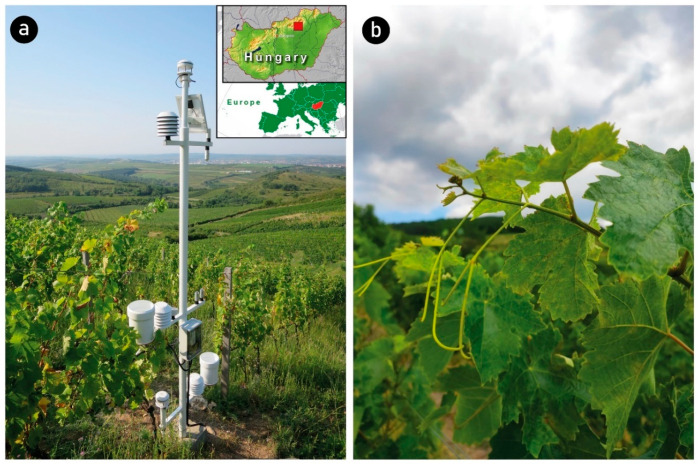
Location of the investigated site in Eger, Hungary. (**a**) Vineyard with data logger equipment; (**b**) grape leaves with drying symptoms.

We believe that the dryness index (DI) developed for grapes is suitable for quantifying this problematic effect. The dryness index is an additive water balance index, described by [12], developed explicitly to quantify climatic water scarcity in grapes. The plant evaporation member of the index contains the crop coefficient function (K_c_) set explicitly for grapes. Drought and the accompanying lack of water trigger a survival mechanism in most plants, as in grapes. Maturation processes are accelerated [13], and berry growth slows or may stop. Due to the smaller-than-average berry size, the solution concentration in the berries will be denser, which usually means a higher sugar content. This higher sugar content occurs mainly when significant crop restrictions are applied in the vineyard. Water scarcity simultaneously reduces the berries’ sugar content and the expected yield amount [14]. The onset of drought simultaneously increases the anthocyanin and tannin content of the berry [15,16,17]. These are favorable conditions for quality grape production, but these advantages are particularly pronounced in the production of red wine [18]. The aroma potential of white grapes decreases due to drought [19]. In a severe drought or water shortage, the berries may lose a significant part of their water content, becoming dried compacted berries without a harvestable crop. Severe drought slows down the process of photosynthesis (Figure 1b) and, thus, the ripening, especially if the yield is high. Both quantitative and qualitative indicators of grape harvest deteriorate under significant drought stress [20]. In many countries, grapes are grown under irrigation, although irrigation can reduce the grapes’ sugar content and other quality indicators. Therefore, the authors of ref. [21] propose a limited amount of irrigation that alleviates the intense water stress state but maintains a slight degree of drought for the quality characteristics of the grapes to develop favorably. In wetter vintages, a larger-than-average berry size usually results in berries with a more dilute solution concentration, meaning the sugar content of the berries decreases. Therefore, the optimum degree of drought is a very narrow range below or above which the grapes do not reach the optimum sugar content of the variety. The planned investigation aims to verify [22]’s statement that grapes have the highest sugar content with moderate water scarcity through the example of some Hungarian grape varieties in the Eger wine region (Figure 1).

The goals of our research are the following:

1. To prove that there is a significant relationship between the dryness index and the fluctuation of the sugar content in wine grapes.
2. To verify the assumption that we can expect maximum sugar content for wine grapes in the case of moderate drought.
3. To determine for which dryness index values we can expect the maximum sugar content to develop.
4. By generating the expected future distribution of the dryness index, it is possible to calculate the probability of maximum sugar content in the near (2016–2035) and distant (2081–2100) future.

## 2. Materials and Methods

### 2.1. Location of the Research Field

The investigations were carried out in the Eger wine region. The 25-hectare Research Institute of Viticulture and Enology of the Eszterházy Károly Catholic University is in the southwestern part of Eger. The name of this cultivation area is Kőlyuktető (geographical location: lat. 47°51′57.2″ N, long. 20°22′51.5″ E).

### 2.2. Examined Wine Grape Varieties

Six wine grape varieties’ (Cabernet Franc, Cabernet Sauvignon, Welschriesling (Riesling Italico), Lemberger, Leanyka (Feteasca Alba), Nero) sugar contents were analyzed during the ripening period. The spur cordon-trained vines with single arms positioned at 2.4 × 1 m inter- and intra-row spacing, respectively. The investigated varieties are of decisive importance in the Eger wine region.

### 2.3. Sugar Content Data

The sugar (g/L) content of the grapes was analyzed in the laboratory of the Food Science and Oenology Knowledge Center of the Eszterházy Károly Catholic University. The sugar sampling data are taken every year at harvest times. The harvest dates are determined based on the ripening dynamics of the grapes. Annual sampling was carried out by randomized block sampling with three replications. The grapes were crushed, and the must sugar concentration was determined from the juice samples based on the method of [23].

The analytical methods recommended by the authors of [24] were used to determine the grapes’ sugar concentration (OIV-MA-AS311-01A). The length of the sugar content time series is different. The most extended series of measurement data was available for the Lemberger between 1987 and 2015. This variety is the most significant in the Eger wine region. At the same time, the shortest data series were available for the Cabernet Franc and Cabernet Sauvignon varieties between 1999 and 2015. The length of the time series of the sugar content database for examined wine grape varieties is shown in Table 1.

### 2.4. Meteorological Database

The DI value was generated using the FORESEE database. FORESEE is a free-access database that currently contains daily data on seven meteorological variables [25]. The seven meteorological variables included in the database are: daily minimum temperature, daily maximum temperature, daily average temperature, daily global radiation, daily precipitation, average daytime temperature, daily vapor pressure deficit.

The ten regional climate models (RCMs) run under the ENSEMBLES European Union project (FP6) are based on state-of-the-art error correction of daily maximum/minimum temperature and precipitation datasets. In the case of precipitation, its time and amount distribution was also corrected. Adjusted climate projections for the future were made using data from the E-OBS past database.

The impact of future human activity was considered according to a medium scenario, A1B SRES (Special Report on Emissions Scenarios; [26]).

The DI values and their distribution functions were determined using ten models’ regional and global forecast climate model data (Table 2). The original grid resolution for all models is the same 0.22° × 0.22°. The database covering the whole of Hungary contains 2070 pixels of data per variable and model, of which the 22 Hungarian wine regions are 300 pixels, and the Eger wine region is 7 pixels.

The results are presented divided into three time periods, based on the averages of the periods corresponding to the IPCC reports [39]. The recent past represents the period 1986–2005. The relative and absolute changes were characterized in the near and distant future compared to this period. The near future is usually characterized by the period 2016–2035, while the distant future is characterized by the period 2081–2100. This approach and time scales will be followed in the study. The calculations assume that the nature of the drought–sugar relationship determined by historical data will also show a similar physiological interaction in the future.

### 2.5. Statistical Analysis

This approach and time scales will be followed in the study. The trend deviations of the sugar content were calculated from the sugar content time series. A detailed description of this linear model approach can be found in [2]. With this method, the trend effect was removed from the sugar content data series. In addition, applying regression analysis can determine the maxima of quadratic regression functions. Furthermore, the distribution and density functions of DI values can be calculated in the near (2016–2035) and distant (2081–2100) future for all investigated wine grape varieties. Assuming it is possible to determine for each grape variety at which DI value maximum sugar content is expected, then, the future development of the sugar content can be estimated for the grape varieties by knowing the future distribution of the DI values.

### 2.6. Dryness Index

The dryness index is an additive water balance index described by [12], developed explicitly to quantify climatic water scarcity in grapes.

$$\mathrm{DI}=\overset{September}{\underset{April}{\sum }}(W_{0}+P-T_{v}-E_{s})$$

The index consists of 4 members, of which members 1 and 2 characterize water intake, and members 3 and 4 represent the expenditure side. Initial (starting as of 1 April) soil moisture stock W_0_ (mm), which most indices calculate [40], fixed in the climatic study of production sites, at W_0_ = 200 mm [41].

1. Initial (starting as of 1 April) soil moisture stock W_0_ (mm), which most indices calculate;
2. Monthly precipitation from 1 April, summed monthly (P (mm));
3. Plant evaporation T_v_ = ET_0_ * K_c_ (mm);
4. Soil evaporation E_s_ = (ET_0_/N) * (1 − K_c_) * JPm (mm).

ET_0_ is the monthly potential evaporation (calculated by the [42] method), and K_c_ is a crop coefficient function. The value of the K_c_ function determined by [41] for the Northern and Southern Hemispheres was defined as follows:

For the Northern Hemisphere, K_c_ = 0.1 in April, K_c_ = 0.3 in May, and K_c_ = 0.5 from June to September.

For the Southern Hemisphere, K_c_ = 0.1 in October, K_c_ = 0.3 in November, and K_c_ = 0.5 from December to March.

N, the sum of the days in a given month, JPm is the number of days with evaporation above 5 mm, obtained by dividing the monthly precipitation (mm) by 5. By definition, JPm ≤ the number of days in a given month N.

The value of the dryness index must not exceed 200, i.e., DI ≤ 200 mm. In the case of the dryness index, the climatic assumption is made that by the end of winter, or, more precisely, by the beginning of the growing season, the soils will be replenished to a minimum water capacity.

## 3. Results

### 3.1. Trend Deviation of Sugar Content Time Series

**Figure 2:**
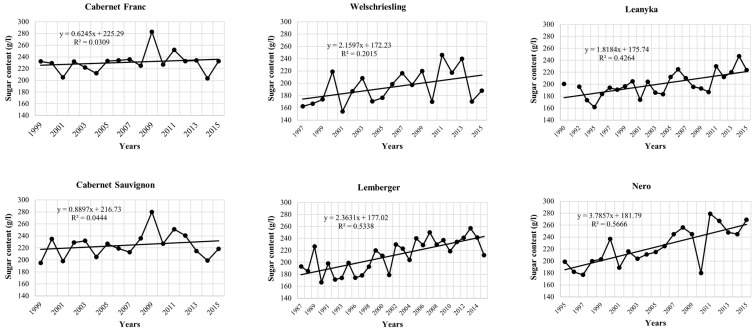
Development of time series and trend functions of sugar content values for six examined wine grape varieties.

The sugar content data series are similar to international trends [43]. The research results show that the sugar content of grapes is increasing yearly (Figure 2).

Since the time series of the sugar content values of the investigated grape varieties show a linearly increasing trend, it is advisable to de-trend the time series, since the trends can have several effects that are difficult to parameterize (soil type, genetics, agrotechnics, canopy development). According to our assumption, environmental parameters, in this case, drought, are usually responsible for positive and negative deviations from the trend function. This linear trend model approach is widely used in agrometeorology practice [44,45]. This linear trend model, was also used in the examination of the trend deviations of the grape wine yield [2].

### 3.2. Relationship between Dryness Index and Sugar Content Trend Deviation

Quadratic relationships were found between the dryness index and the trend deviation values of sugar content for all wine grape varieties. Since the square prefix of the quadratic functions is negative, this means that the functions have a maximum, which means that at a certain DI, maximum sugar content (SCmax) is expected.

**Figure 3:**
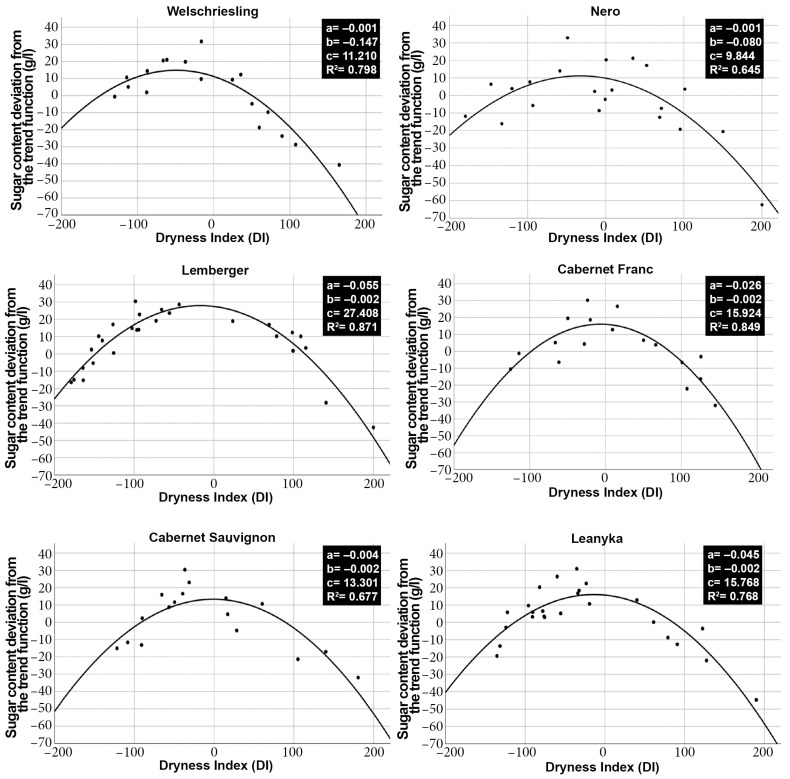
Regression relationships between the dryness index and the sugar content of the six grape varieties studied. Coefficients and significances of quadratic regression equations are highlighted.

The R^2^ coefficient of the regression relationship between DI and sugar content was the highest (R^2^ = 0.871) for Lemberger. At the same time, the lowest value (R^2^ = 0.645) was observed for Nero. In all six grape varieties, there was a significant quadratic regression relationship between the dryness index and sugar content deviation from the trend (Figure 3). The quadratic equation can be expressed as follows:

$$y=ax^{2}+bx+c$$

The derivation process of the quadratic regression relationship can be described by the following equations:

$$y\left(DI\right)=a\;DI^{2}+b\;DI+c$$

$$y^{\prime }\left(DI\right)=2\;a\;DI+b$$

$$y^{\prime }\left(DI\right)=0\;\left\{y_{max}\;;\;y_{min}\right\}$$

$$\frac{-b}{2\;a}=DI\;\left\{y_{max}\right\}\;$$

**Figure 4:**
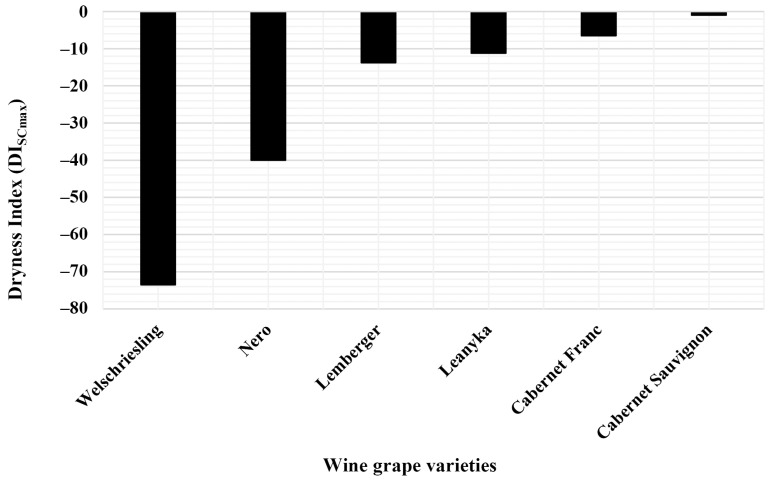
Calculated dryness index values for the maximum sugar content of the examined grape varieties. DI_SCmax_ values are required to reach the maximum sugar content for the six grape varieties represented in the column function.

Knowing the derivative functions, it could be determined at which DI values the maximum sugar content (SCmax) occurs for each wine grape variety. Figure 4 shows that the maximum sugar content (SCmax) values occurred in the case of small negative DI index values for all investigated wine grape varieties. The degree of drought required to achieve the maximum sugar content (SCmax), known after this as the optimal drought, differs significantly for the examined grape varieties. With this DI measure, the drought tolerance of wine grape varieties can also be numerically characterized. Supposing a grape variety can produce a maximum sugar content with an even more significant drought, it is presumably more tolerant of drought than varieties with maximum sugar content with only slight dryness.

The results show that Welschriesling has the highest drought tolerance of the grape varieties studied. Nero followed it, which also had an outstandingly good drought tolerance. The results show that the drought tolerances of Lemberger and Leanyka were nearly identical but significantly lower than those of Welschriesling or Nero. Cabernet Franc and Cabernet Sauvignon show the lowest drought tolerance among the tested wine grapes (Figure 4).

### 3.3. Future Changes of Optimal Drought Probabilities

**Figure 5:**
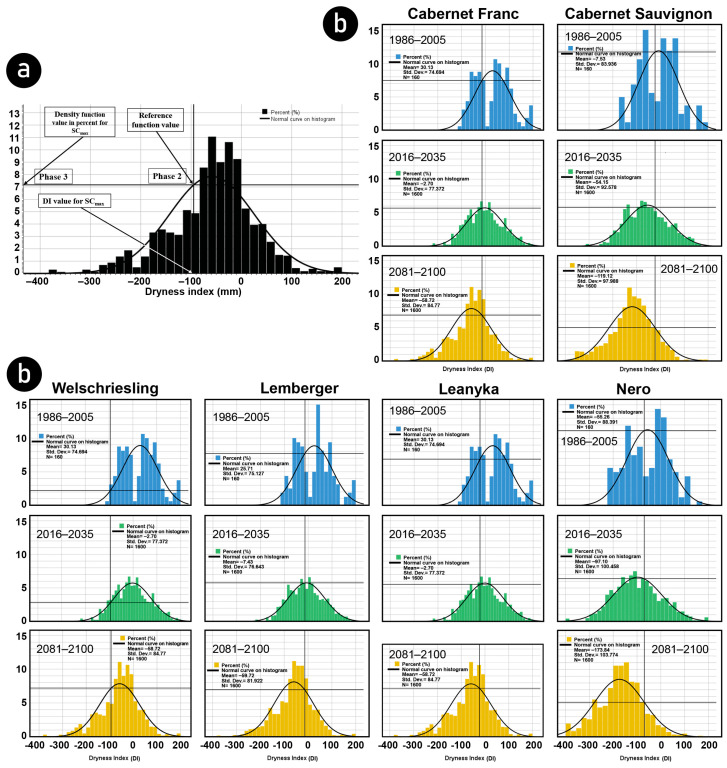
Determination of the probability value of optimal drought occurrence. (**a**) Steps for determining the probability value of the optimal drought occurrence. (**b**) The dryness index and DISCmax of the six examined grape varieties distributed in three IPCC periods.

The probabilities of optimal drought occurrence can be investigated by determining the distribution functions of the DI values for the IPCC periods of a given test site. The density function for a given distribution function is then defined. The probability of optimal drought given as a percentage can be determined in three steps, as shown in Figure 5a. The first step is to fit a straight line to the DI value for SCmax (Phase 1); this vertical line intersects the density function, and it will be the reference function value (Phase 2). The probability is determined by fitting a horizontal line to the reference function value and reading the probability value in % on the vertical axis (Phase 3). Based on the above method, the optimal drought probability value can be calculated for each examined grape variety for both the near and far IPCC periods (Figure 5b).

Supposing a wine grape variety currently grown is likely to have maximum sugar content in the future, this is generally a favorable growing condition, as a high sugar content (SC_max_) is a prerequisite for producing quality wine. Supposing the weather conditions have been favorable for the production of quality wine in the past, meaning there is no or scarce vintages where the sugar content of the grapes would have been lower than necessary. In that case, the increase in the years providing maximum sugar content (SC_max_) is unfavorable in a wine region. Supposing an excellent quality wine can be produced in the case of vintages with a high sugar content (SCmax), an increase in the incidence of optimal drought is favorable for growers; if the high sugar content results in a disproportionately high alcohol content in the wines, the probability of an optimal drought increase can be considered unfavorable.

The standard deviation of the probability of optimal dryness occurrence of the studied cultivars was recently 3.4. In contrast, the relative standard deviation reached 43.2%. Except for the Welschriesling variety, the probability of optimal drought occurrence will decrease in the near future. In addition, the standard deviation (1.3) and relative standard deviation (24%) of the probability values are significantly reduced. Smaller drought probabilities and smaller drought variations will characterize the decade ahead. It is good news for producers worried about wines with higher alcohol content than they need. In the distant future, however, the probability of optimal drought increases for most of the cultivars studied, except for Nero and Cabernet Sauvignon, where the incidence of optimal drought decreases further, while for Welschriesling, the probability of optimal drought increases in both the near and distant future (Table 3).

**Figure 6:**
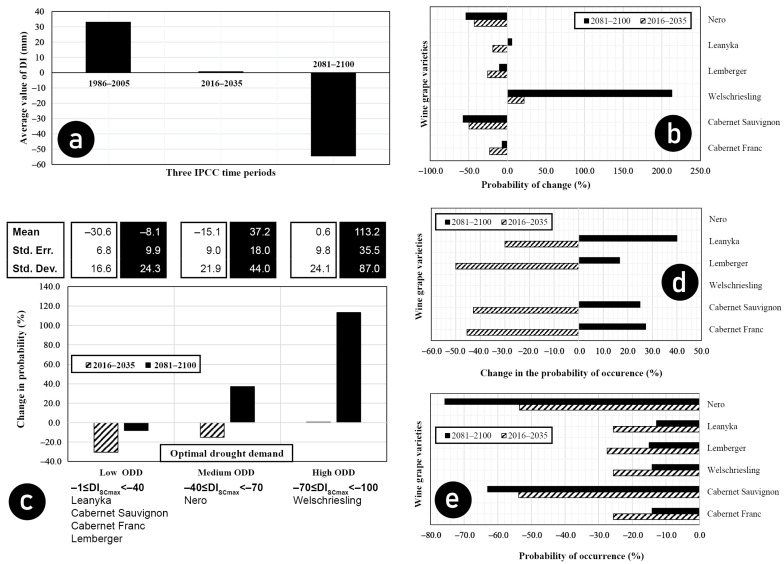
Future predictions. (**a**) Changes in the average value of the dryness index in the recent past and near and distant future; (**b**) changes in the probability of optimal drought in percentages, compared to the period 1986–2005; (**c**) changes in the occurrence of optimal drought demand compared to the period 1986–2005. Optimal drought demand (ODD) categories and their associated DI_SCmax_ intervals and grape varieties and changes in the averages, errors and standard deviations of the optimal drought demand interval in the near and distant future as a percentage of the average of the period 1986–2005 are detailed; (**d**) changes in the probability of optimal drought compared to 1986–2005 in percentage values; (**e**) changes in the likelihood of the occurrence of moderate drought compared to the period 1986–2005 in percentages.

The results show that except for Welschriesling, the probability of optimal drought occurring in the near future—between 2016 and 2035—decreases for all studied varieties. In the distant future, between 2081 and 2100, except for Nero and Cabernet Franc, the probability of the occurrence of maximum sugar content will increase again (Figure 6b).

### 3.4. Simulated Deficit Irrigation

The deficit irrigation simulation was based on the dryness index (DI). A positive DI range means an excess of water. In contrast, in the case of a small negative dryness index range, we can speak of a moderate water deficit.

Test results show that the degree of drought will increase in the future. Our results show that the average DI value will reach −50 mm by the end of the century (Figure 6a). This change poses a severe challenge to producers. Providing moderate drought for the wine grape varieties grown continuously will be challenging. More than optimal dryness reduces the sugar content and the yield amount of grapes that can be harvested. The solution is to apply irrigation to maintain moderate drought for current grape varieties or introduce drought-tolerant varieties in the given production area.

Let us see how the optimal drought incidence would develop if we did not allow the DI to drop below −10 mm using water replacement irrigation. Due to the simulated deficit irrigation, the distribution and density functions previously defined for the varieties are distorted, as there will be no DI values less than −10 in the sample.

In the case of the Welschriesling and Nero cultivars, the results cannot be interpreted because the DI_SCmax_ value characteristics of the cultivars is outside the range of interpretation of the distribution functions, especially in the near and distant future. Regulated deficit irrigation can only protect against rising sugar in the near future, while in the distant future, there will be an increase in all wine grape varieties examined (Figure 6d). The effect of regulated deficit irrigation on sugar content is mainly short-term. In the near future, especially for the Cabernet Franc, Lemberger and Leanyka varieties, it is observed that the probability of the maximum sugar content characteristic of the wine grape variety decreases to a greater extent than in the case of non-irrigation. Deficit irrigation that maintains moderate drought can effectively lower the sugar content for these varieties. However, the regulated deficit irrigation will not provide sufficient protection against the increase in sugar content due to increasing drought in the distant future.

### 3.5. Optimal Drought Requirements of Varieties

The studied varieties can produce maximum sugar content with different degrees of drought. After this, the drought value, more precisely the DI value at which the maximum sugar content is obtained for a given grape variety, is called the optimal drought demand (ODD). Based on the DI_SCmax_ values of the studied cultivars, we can talk about cultivars with low, medium and high ODD requirements. Low ODD cultivars were characterized by lower negative DI_SCmax_ values. High ODD cultivars were characterized by higher negative DI_SCmax_ values (Figure 6c).

Let us analyze the previously determined DI distributions for each wine grape variety and determine the optimal drought requirements of each wine grape variety. Examining how the low (DI_SCmax_ = −25), medium (DI_SCmax_ = −55), and high ODD values (DI_SCmax_ = −85) change the probability values of maximum sugar content distribution functions in the near and distant future, we obtain the following results.

The probability values of the maximum sugar content of low ODD varieties will decrease sharply in the near future but moderately in the distant future. In the case of medium ODD varieties, a moderate decrease can be expected in the short term. In contrast, a significant increase in the maximum sugar content can be expected in the long term. For varieties with high ODD, no change is expected in the near future. In contrast, in the distant future, we can expect a significant increase in the probabilities of maximum sugar content (Figure 6c).

The errors and standard deviations of the means of the ODD intervals vary inversely with the ODD values of the varieties. The smaller the negative ODD value characterizing a given wine grape variety, the greater the mean error and standard deviation. Examining the changes in the near and distant future compared to the average of the base period 1986–2005, we can expect more significant errors and more extensive standard deviations in the distant future than in the near future (Figure 6c).

If all varieties had a uniformly low ODD value, meaning if DI_SCmax_ = −10 were satisfied, the probability of maximum sugar content would be significantly reduced both in the near and distant future compared to the recent past (Figure 6e). The reduction rate would be most significant for the Nero and Cabernet Sauvignon varieties. For these varieties, the probability of the occurrence of the maximum sugar content would already decrease by 50% in the near future, and we could expect a decrease of more than 70% in the distant future.

## 4. Discussion

In connection with climate change, the changes in circulation conditions and the development of a more frequent blocking situation have been reported by [46], as well as the precipitation extremes and drought that can be expected to increase in the future in our area [47]. The European continent, particularly the Carpathian Basin, has exceptionally diverse climatic conditions.

The selection of cultivation areas for vineyards is an essential task for vine growers. Those grapes (*Vitis vinifera* L.) grown under favorable water supply conditions usually show excessive growth with low-quality produce, while grapes with weak growth result in low yields and their cultivation is uneconomical [48]. Abundant water supply, which can often occur in vineyards with poorly regulated irrigation systems, significantly increases grape growth, increases leaf area [49,50] and increases shoot growth [51,52]. The increased foliage has a more significant shading effect, which prolongs the ripening process and thus reduces the quality parameters of the berries [53]. Excessive irrigation creates competition between shoot and berry growth [54]. As a result, the sugar content of grapes decreases [55,56]. These test results are consistent with the results we obtained. For dryness index values above +100, the sugar content of the grapes decreases significantly. A high positive dryness index means that the environment and the vineyard area have an abundant water supply. As a result, there is an increased likelihood that shoot growth will be strong and a dense canopy will form. Supposing the high positive dryness index remains characteristic throughout the growing season, shoot growth may remain active throughout the growing season. Excessive shading develops due to the numerous leaves [57,58,59]. The increased shadow effect reduces the quality content of the berry [60], i.e., the sugar content of the berries decreases [61,62]. Therefore, it is unsurprising that compared to most cultivated plant species, grapes are grown in a geographical and climatic environment where the water supply is optimal, i.e., there is moderate water stress during the growing season to improve wine grape quality. Several authors have addressed the effect of water scarcity and water supply on sugar content [63,64,65,66,67]. However, the effect of the dryness index on the development of the sugar content of wine grapes has not been studied so far. Most authors agree that berry quality indexes depend on the severity and duration of dryness and the phenological condition of the grapes. Most studies have analyzed the effect of different amounts of irrigation water on the grape quality contents [68,69,70]. There is widespread agreement that a moderate water deficit increases the sugar content of grapes [71,72,73], partly due to a decrease in berry size and partly due to an increased ripening rate. These previous studies confirm our results that we can expect a maximum sugar content in the range of −13 < DI_SCmax_ < −95. This range can be considered moderate water scarcity for the dryness index. The occurrence, extent, and timing of mild water scarcity are significant factors in the “terroir” effect [74,75]. The use of regulated deficit irrigation, developed to create timed and controlled water stress, is an excellent help for vine growers in creating controlled water stress [76]. We believe the dryness index is also suitable for determining moderate water stress status. If we know the spatial distribution of the dryness index, the geographical environment in which the climatic conditions are favorable for the cultivation of wine grapes can be determined precisely in the future. While mild water stress primarily affects cell turgor, severe water scarcity also negatively affects other physiological processes such as photosynthesis or solute transport [77]. Severe water deficit reduces assimilated production, evaporation, shoot growth, yield, and fruit quality [78,79,80]. Severe water deficit after veraison significantly decreases the accumulation of sugar. As a result, the ripening process may collapse, and the berries remain unripe with low sugar content [81]. These results are also consistent with the studies we obtained, i.e., in the case of significant water scarcity, when the dryness index is below −100, the sugar content of the berries decreases significantly.

We believe that introducing optimal drought demand (ODD) to growers can prove helpful if it is known for each cultivated variety. It will be possible for growers to implement deficit irrigation on a variety-specific basis in the future. Based on the above, we believe that the dryness index values we modified are suitable for future estimation of the probability of maximum sugar content. Assuming our goal is to reach maximum sugar content, we should strive to keep the dryness index values for the growing area in a small negative value range during the growing season, depending on the variety. If there is a severe drought, we can keep the dryness index values within the appropriate ranges with regulated deficit irrigation. Supposing that the value of the dryness index is in the higher positive range in the given vintage, then, achieving maximum sugar content with the traditional cultivation technology methods is impossible. As an extremely high sugar content is a significant problem in many winegrape cultivation areas, deficit irrigation can solve this problem.

## 5. Conclusions

Our studies confirmed the statement of [22] that the sugar content of grapes is at its highest with moderate drought. For the six grape varieties, we examined the dryness index (DISCmax) value used by us to obtain the maximum sugar content between −13 and −95. It is difficult to define precisely the DI value of moderate drought. In the [41] dryness index (DI)-based site classification, areas with moderately dry climatic conditions are characterized by dryness index values between −100 and +50. The mainly arid regions are characterized by DI ≤ −100.

Considering this, the DI_SCmax_ values that we calculated were required to form the maximum sugar content can be regarded as the DI value of moderate drought.

Based on the results, it is necessary to rethink the varieties that can be grown for the given production area in the future. There will be varieties that will be less able to adapt to increased drought and a higher heat sum. The results show that in the case of the Eger wine region, the cultivation of Cabernet Sauvignon and Nero varieties could avoid excessive sugar content in the future. However, the Riesling and the Leanyka varieties may have future sugar content problems. Irrigation, which helps maintain a moderate drought, can be a helpful way to regulate sugar content in viticulture in an environmentally friendly way, especially in the short term, meaning in the near future. In the distant future, this method will not reduce the probability of maximum sugar content. The extent to which drought-maintaining irrigation is required to reduce the likelihood of maximum sugar content may be further investigated. The other option is to grow “climate-tolerant” grape varieties well adapted to increased dryness and higher heat. Depending on whether we want to reduce or increase the probability of maximum sugar content in the future when growing a given variety, growers have to choose a cultivar with a different optimal drought demand. Supposing we want to reduce the sugar content, then, selecting a variety with a low ODD is advisable, so the probability values of maximum sugar content will decrease both in the near and distant future. Assuming we want to increase the chances of maximum sugar content in the future, then, choosing a variety with a high ODD is advisable. In this case, although the probability of the occurrence of maximum sugar content will not increase significantly in the near future, we can expect a significant increase in the distant future.

## Figures and Tables

**Table 1:** The length of time series of the sugar content database for the six analyzed wine grape varieties.

Wine Grape Varieties	Length of Time Series
Cabernet Franc	1999–2015
Cabernet Sauvignon	1999–2015
Leanyka	1990–2015
Lemberger	1987–2015
Nero	1995–2015
Welschriesling	1997–2015

**Table 2:** Regional-global forecast climate models used in the Foresee database.

Regional Climate Models (RCM)	General Circulation Models (GCM)	Institution	References
ALADIN	ARPEGE	Centre National de Recherches Météorologiques	[27]
CLM	HadCM3Q0	Eidgenössische Technische Hochschule. Zürich	[28,29]
HadRM3Q0	HadCM3Q0	Hadley Centre	[30]
HIRHAM5	ARPEGE	Danish Meteorological Institute	[31]
HIRHAM	ECHAM5	Danish Meteorological Institute	[31]
RACMO2	ECHAM5	Koninklijk Nederlands Meteorologisch Instituut	[32]
RCA	ECHAM5	Sweden’s Meteorological and Hydrological Institute	[33,34]
RCA	HadCM3Q3	Sweden’s Meteorological and Hydrological Institute	[33,34]
RegCM3	ECHAM5	International Centre for Theoretical Physics	[35,36]
REMO	ECHAM5	Max Planck Institute	[37,38]

**Table 3:** The probability of optimal drought occurring in the three IPCC periods and for six grape varieties and changes in the probability values of the optimal drought with regulated deficit irrigation (DI_min_ ≥ −10) for six grape varieties in the three IPCC periods.

Wine Grape Varieties	1986–2005	2016–2035	2081–2100
	DI_Scmax_ (%)	DI_Scmax_ (%)	DI_Scmax_ (%)
Cabernet Franc	7.4	5.7	6.9
Cabernet Sauvignon	11.7	5.9	5
Welschriesling	2.3	2.8	7.2
Lemberger	7.8	5.8	7
Leanyka	6.9	5.6	7.3
Nero	11.2	6.4	5.2
Mean	7.9	5.4	6.4
Standard deviation	3.4	1.3	1
Relative standard deviation (%)	43.2	24	16.2

## Data Availability

The datasets generated during and/or analyzed during the current study are available from the corresponding author upon reasonable request.
